# The Control of Tendon-Driven Dexterous Hands with Joint Simulation

**DOI:** 10.3390/s140101723

**Published:** 2014-01-20

**Authors:** Jinbao Chen, Dong Han

**Affiliations:** College of Astronautics, Nanjing University of Aeronautics and Astronautics, Nanjing 210016, China; E-Mail: han_dongnuaa@126.com

**Keywords:** adaptive impedance control, tendon-driven dexterous hand, joint simulation

## Abstract

An adaptive impedance control algorithm for tendon-driven dexterous hands is presented. The main idea of this algorithm is to compensate the output of the classical impedance control by an offset that is a proportion-integration-differentiation (PID) expression of force error. The adaptive impedance control can adjust the impedance parameters indirectly when the environment position and stiffness are uncertain. In addition, the position controller and inverse kinematics solver are specially designed for the tendon-driven hand. The performance of the proposed control algorithm is validated by using MATLAB and ADAMS software for joint simulation. ADAMS is a great software for virtual prototype analysis. A tendon-driven hand model is built and a control module is generated in ADAMS. Then the control system is built in MATLAB using the control module. The joint simulation results demonstrate fast response and robustness of the algorithm when the environment is not exactly known, so the algorithm is suitable for the control of tendon-driven dexterous hands.

## 1. Introduction

The control system of dexterous robot hands can control the robot fingers to reach the desired position, contact with the environment and track a specified desired force. Thereinto, stable force tracking is an important research topic, which can be solved by compliance control. Impedance force control is very practical in the field of robotic compliance control and the main concept is based on the impedance equation which is the relationship between force and position/velocity error [1].

Many researchers have improved the performance of the impedance control and expanded the application range since it was primarily proposed by Hogan [2–5]. However, the classical impedance control is unsatisfying when the environment parameters are not exactly known. To overcome this problem, Lasky *et al*. [6] proposed a two-loop control system that the inner-loop is a classical impedance controller and the outer-loop is a trajectory modified for force-tracking. This algorithm uses the outer-loop to automatically modify the reference position by a simple force-feedback scheme when the environment is not exactly known. Jung *et al*. [1] proposed an adaptive impedance control. The main idea of this algorithm is to minimize the force error directly by using a simple adaptive gain when the environment is changed. Seraji [7] proposed an adaptive admittance control based on the concept of mechanical admittance, which relates the contact force to the resulting velocity perturbation. Two adaptive PID and PI force compensators are designed in Seraji's paper.

In this paper an adaptive impedance control is proposed that uses an adaptive PID force compensator as an offset to adjust the output of the impedance controller when the environment position or stiffness is changed. It is a way that adjusts the impedance parameters indirectly, which is different from Jung's. In order to validate the algorithm, a joint simulation with MATLAB and ADAMS is presented. Firstly, the model of the tendon-driven dexterous hand is built in ADAMS referring to the robot hand of Robonaut-2, which is the first humanoid robot in space and has the typical tendon-driven dexterous hands [8]. A three-DOF finger of the robot hand is chosen as the research object. Then a control module of the robot finger is generated in ADAMS. Finally, the control system is built in MATLAB using the control module. The results of the joint simulation demonstrate that the proposed algorithm is robust. In addition, the position controller and inverse kinematics solver are designed for the tendon-driven finger.

## 2. Features of the Robot Hand and Dynamic Model

**Figure 1.**
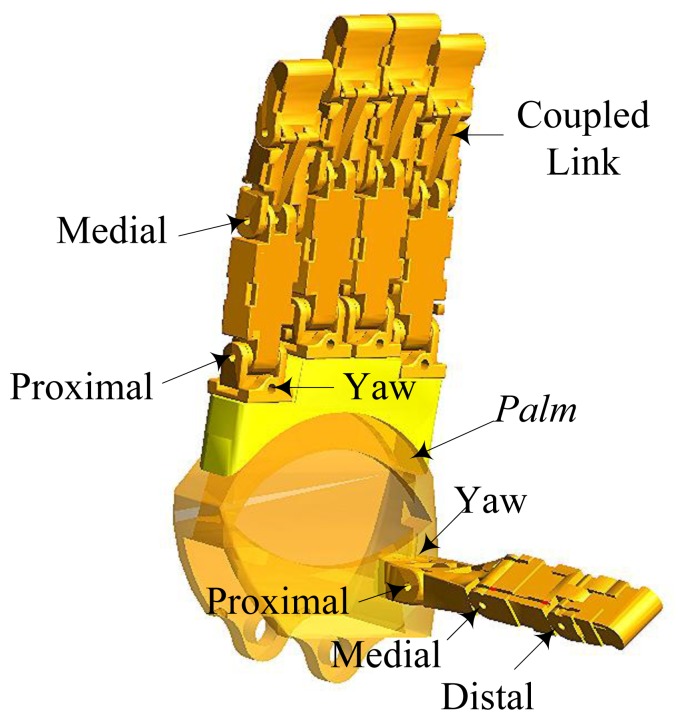
The model of the dexterous hand in ADAMS.

The model of the tendon-driven dexterous hand in ADAMS consists of four three-DOF fingers, a four-DOF thumb and a palm, as shown in Figure 1. For the three-DOF fingers, the fingertip's motion depends on the coupled link, as shown in Figure 1. The actuation system of the robot hand is remotely packaged in the forearm, which makes the size of the robot hand as large as a man's hand. Each unit of the actuation system consists of a brushless motor and a lead screw. The lead screw can convert rotary motion to linear motion. Each of the tendons connects the finger joint and the lead screw. The motors can drive the lead screws to control the finger motion through the tendons. Since the tendons can only transmit forces in tension, the number of tendons should be more than the DOFs. It turns out that only one tendon more than the number of DOF is needed [9], so a three-DOF finger needs four tendons.

The dynamic model of the three-DOF finger can be expressed as follows:

(1) $$M(q)\ddot{q}+C(q,\dot{q})\dot{q}+g(q)=\tau ‐\tau_{\text{f}}+\tau_{\text{ext}}$$

where ***M***(***q***), ***C***(***q***) and ***g***(***q***) represent the inertia matrix, centrifugal term, and gravity term respectively; ***q*** represents the joint angle vector; ***τ***, ***τ***_f_ and ***τ***_ext_ represent the joint torque vector, friction torque vector and external torque vector respectively. ***τ***_ext_ is given by [10]:

(2) $$\tau_{\text{ext}}=J^{T}F_{t}$$

where ***J*** represents the Jacobian matrix, ***F***_t_ = [ ***F***_tx_ ***F***_ty_ ***F***_tz_]^T^ represents the fingertip force. ***τ***_m_ is defined as the motor torque vector. Then there should be a certain relationship between ***τ*** and ***τ***_m_, which is shown as follow.

**Figure 2.**
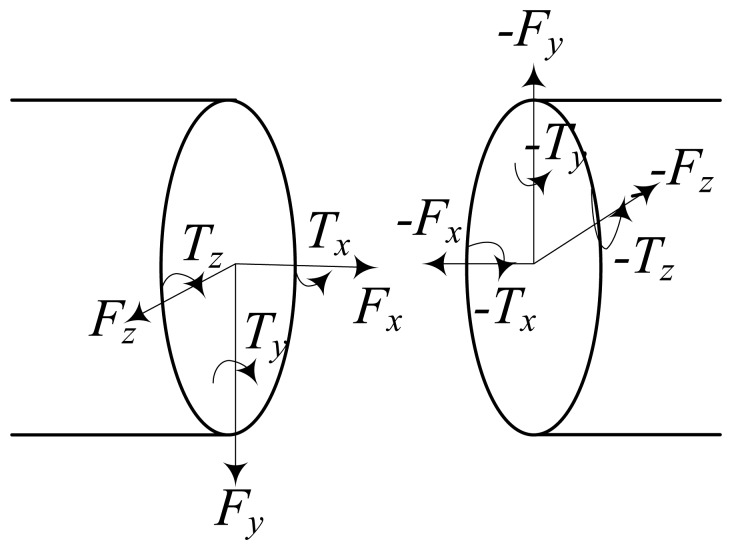
Force analysis between a two small cylinders of the tendon model.

Firstly, for the three-DOF finger, the transformation from four tensions to three joint torques is given by [9,10]:

(3) $$\tau =Rf,R=\left[\begin{array}{cccc} r_{11} & r_{12} & -r_{13} & -r_{14}\\ r_{21} & -r_{22} & r_{23} & -r_{24}\\ 0 & 0 & r_{33} & -r_{34} \end{array}\right]$$

where ***f*** is a column vector consisting of four tendon tensions; ***R*** represents the mapping from tensions (***f***) to joint torques (***τ***) and ***r****_ij_* is the radius of the circular surface where the *j*-tendon envelops itself on the *i*-joint (The tendons are numbered from *t*_1_ to *t*_4_, as shown in Figure 2), *i* = 1 ∼ 3, *j* = 1 ∼ 4. For the three-DOF finger, *r*_11_ = *r*_12_ = *r*_13_ = *r*_14_ = 5.5 mm, *r*_21_ = *r*_22_ = *r*_23_ = *r*_24_ = 5.2 mm, *r*_33_ = *r*_34_ = 5.0 mm.

Secondly, the relationship between the tendon tensions and motor torques is expressed as:

(4) $$f=\frac{2\pi }{d}\tau_{\text{m}}$$

where *d* represents the screw pitch of the lead screws.

According to Equations (3) and (4), the Equation (1) can be rewritten as:

(5) $$\text{M}(\text{q})\ddot{q}+\text{C}(\text{q},\dot{\text{q}})\dot{q}+\text{g}(q)=\frac{2\pi }{d}R\tau_{\text{m}}‒\tau_{\text{f}}+\tau_{\text{ext}}$$

The above indicates that the tendons have an impact on the control of the tendon-driven robot hand. In order to simulate the performance of the tendons in ADAMS, the tendon model is built by adding bushing force between small stiffness cylinders. The force analysis between a two small cylinders is shown in Figure 2.

**Figure 3.**
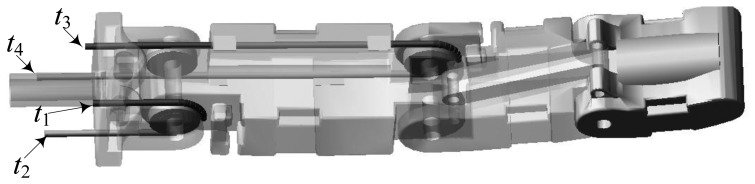
The tendon model in the three-DOF finger.

The dynamic equation of the tendon model can be expressed as [11]:

(6) $$\left[\begin{array}{c} F\\ T \end{array}\right]=K\left[\begin{array}{c} R\\ \theta \end{array}\right]-C\left[\begin{array}{c} V\\ \omega \end{array}\right]+\left[\begin{array}{c} F_{0}\\ T_{0} \end{array}\right]$$

where ***F*** and ***T*** represent the force and torque between the cylinders respectively; ***K*** and ***C*** represent the stiffness diagonal matrix and damping diagonal matrix respectively; ***R***, ***θ***, ***V*** and ***ω*** represent the relative displacement, angle, velocity and angular velocity between the two cylinders respectively; ***F***_0_ and ***T***_0_ are respectively the initial values of the force and torque. In order to get a reasonable tendon model, ***K*** and ***C*** should be set to appropriate values. In the control of the robot hand, the tendons that are not flexible enough will affect the finger motion but the very flexible tendons will diminish the control accuracy. Considering that, the satisfying tendon models are built by adjusting the parameters of ***K*** and ***C*** in ADAMS [12], as shown in Figure 3.

## 3. The Control System of Tendon-Driven Dexterous Finger

### 3.1. The Components of the Control System

**Figure 4.**
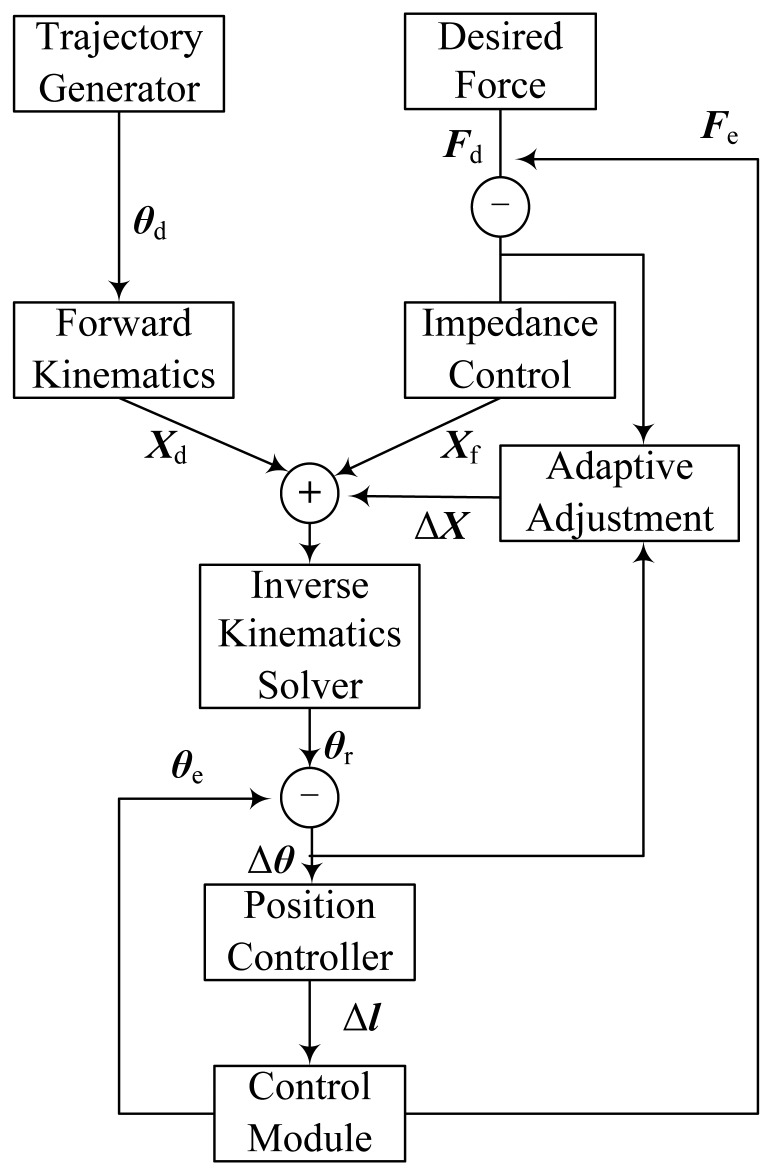
The components of the control system.

The control system is built for the three-DOF finger. It consists of a control module, adaptive impedance controller, trajectory generator, inverse kinematics solver, position controller and other modules as shown in Figure 4.

**Figure 5.**
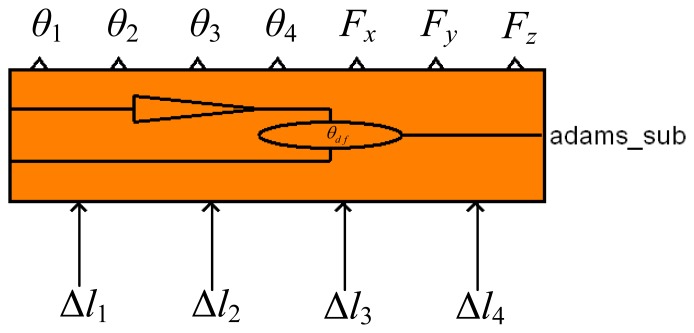
The control module of the three-DOF finger in MATLAB.

The trajectory generator provides the desired angular displacement ***θ***_d_, which need to be converted to the desired displacement ***X***_d_ in Cartesian-space by forward kinematics because the output of the adaptive impedance controller is in Cartesian-space. The adaptive impedance controller consists of an impedance control part and an adaptive adjustment part. The input of the impedance control part is the difference between the desired contact force ***F***_d_ and the actual contact force ***F***_e_ and the output is a displacement offset ***X***_f_. And Δ***X*** is defined as the output of the adaptive adjustment part. ***X***_r_ = ***X***_d_ + ***X***_f_ + Δ***X*** represents the displacement that has been adjusted in Cartesian-space. ***X***_r_ can be converted to the angular displacement ***θ***_r_ in joint-space by inverse kinematics. Δ***θ*** represents the difference between ***θ***_r_ and the actual angular displacement ***θ***_e_. The position controller can convert Δ***θ*** to the tendon displacement offset Δ***l***. The control module is generated in ADAMS. It is the interface between ADAMS and MATLAB and contacts the robot model in ADAMS with the control system in MATLAB. So it can be seen as a MATLAB module that has the same effect with the model in ADAMS. The control module of the three-DOF finger is shown in Figure 5 (by the way, ***θ***_d_, ***θ***_e_, ***θ***_r_, Δ***θ*** and Δ***l*** are four-dimensional column matrices; ***X***_d_, ***X***_f_, ***X***_r_, Δ***X***, ***F***_d_ and ***F***_e_ are three-dimensional column matrices.)

In Figure 5, Δ*l*_1_, Δ*l*_2_, Δ*l*_3_ and Δ*l*_4_ are the input variables of the control module and represent the displacement offset of the four tendons; *θ*_1_, *θ*_2_, *θ*_3_, *θ*_4_, *F_x_*, *F_y_* and *F_z_* are the output variables, where *θ*_1_, *θ*_2_, *θ*_3_ and *θ*_4_ represent the angular displacements of joints and *F_x_*, *F_y_* and *F_z_* represent the components of the contact force.

### 3.2. Adaptive Impedance Controller

The impedance control is based on the concept that it is neither position nor force that should be controlled, but rather the dynamic relation between the two [13]. The relation is an impedance equation, which is given by:

(7) $${\text{M}}_{\text{d}}({\ddot{X}}_{\text{r}}-{\ddot{X}}_{\text{d}})+{\text{B}}_{d}({\dot{X}}_{r}-{\dot{X}}_{d})+K_{d}(X_{r}-X_{d})=E$$

where ***E*** = ***F***_d_ − ***F***_e_; ***M***_d_, ***B***_d_ and ***K***_d_ are respectively 3 × 3 constant-positive-diagonal matrices of the desired inertial, damping and stiffness. The impedance parameters ***M***_d_, ***B***_d_ and ***K***_d_ should keep the system in the critical damping state or overdamping state at least. And increasing ***M***_d_ will result in large impact to environment. A large ***B***_d_ can increase the response time and have no vibration. For position accurate tracking, a large ***K***_d_ should be selected [14]. According to these, the suitable impedance parameters for specified control system can be got.

For a classical impedance control, Δ***X*** = 0 and ***X***_f_ = ***X***_r_ − ***X***_d_. So, Equation (7) can be expressed in the Laplace domain as follows:

(8) $$X_{\text{f}}(s)=\frac{E(s)}{M_{d}s^{2}+B_{d}s+K_{d}}$$

**Figure 6.**
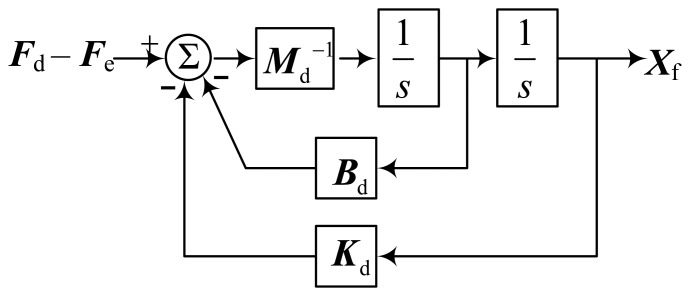
The structure diagram of the impedance control compensator.

According to Equation (8), the structure diagram of the impedance control compensator is shown in Figure 6. However, the impedance control compensator is only suitable for a specific environment and the impedance parameters can't be changed in the whole control process. If the environmental parameters changed or weren't exact, the performance of the controller would be unsatisfactory. Hence an adaptive adjustment is needed to change the impedance parameters indirectly according to the environment changes.

The key of the adaptive adjustment is to get an adaptive offset Δ***X*** to compensate the output of the impedance control. Let Δ*x_k_*, *f*_d_*_k_*, *f*_e_*_k_*, *x*_e_*_k_* and *x*_d_*_k_* respectively represent the *k*-th element of Δ***X***, ***F***_d_, ***F***_e_, ***X***_e_, ***X***_d_ (*k* = 1–3, ***X***_e_ represents the actual position of the fingertip in Cartesian-space). Then Δ*x_k_* can be expressed as follows [7]:

(9) $$\Delta x_{k}=g(t)+k_{p}(t)e(t)+k_{d}(t)\dot{e}(t)$$

where *g*(*t*) is an auxiliary signal which contains the integral term; *k_p_*(*t*) and *k_d_*(*t*) are respectively the adaptive proportional and derivative force feedback gain; *e*(*t*) = *f*_d_*_k_* − *f*_e_*_k_*. Equation (9) is a PID expression of force error in nature, so this adaptive adjustment compensator can respond quickly to the change of the force error, that is to say, to the change of the environment. It is the reason why this expression is used as the compensator of impedance control. *g*(*t*), *k_p_*(*t*) and *k_d_*(*t*) are given by:

(10) $$\begin{array}{c} q(t)=\omega_{p}e(t)+\omega_{d}\dot{e}(t)\\ g(t)=g_{0}+\alpha_{1}\int_{0}^{t}q(t)dt+\alpha_{2}q(t)\\ k_{p}(t)=k_{p0}+\beta_{1}\int_{0}^{t}q(t)e(t)dt+\beta_{2}q(t)e(t)\\ k_{d}(t)=k_{d0}+\gamma_{1}\int_{0}^{t}q(t)\dot{e}(t)dt+\gamma_{2}q(t)\dot{e}(t) \end{array}$$

where *ω_p_* and *ω_d_* are the positive position and velocity weighting factors respectively; *α*_1_, *β*_1_ and *γ*_1_ are the positive integral adaptation gains respectively; *α*_2_, *β*_2_ and *γ*_2_ are the positive or zero proportional adaptation gains. Let *α*_2_, *β*_2_ and *γ*_2_ equal to zero to simplify the expressions. And *k_d_*_0_, *k_p_*_0_ and *g*_0_ are the initial values and equal to zero. Since the actual contact force is measured by the force sensor, there is often a noisy signal. Therefore the derivative of the force error is unsatisfying. To solve this problem, *ė*(*t*) is replaced by −*k_e_ẋ*(*t*) in Equations (9) and (10), where *x*(*t*) = *x*_d_*_k_* − *x*_e_*_k_* and *k_e_* is a positive constant. In addition, *σ*-modification terms are used to modify the adaptive compensator to ensure the robustness of the control system [7,15]. Finally the modified expressions of the compensator are given by:

(11) $$\begin{array}{c} \Delta x_{k}=g(t)+k_{p}(t)e(t)-k_{v}\dot{x}(t)\\ q(t)=\omega_{p}e(t)-\omega_{v}\dot{x}(t)\\ g(t)=g_{0}+\alpha_{1}\int_{0}^{t}q(t)dt-\sigma_{1}\int_{0}^{t}g(t)dt\\ k_{p}(t)=k_{p0}+\beta_{1}\int_{0}^{t}q(t)e(t)dt-\sigma_{2}\int_{0}^{t}k_{p}(t)dt\\ k_{v}(t)=k_{v0}-\lambda_{1}\int_{0}^{t}q(t)\dot{x}(t)dt-\sigma_{3}\int_{0}^{t}k_{v}(t)dt \end{array}$$

where *λ*_1_ = *γ*_1_*k_e_*, *ω_v_* = *ω_d_k_e_*; *σ*_1_, *σ*_2_ and *σ_3_* are small positive constants.

### 3.3. Inverse Kinematics Solver

**Figure 7.**
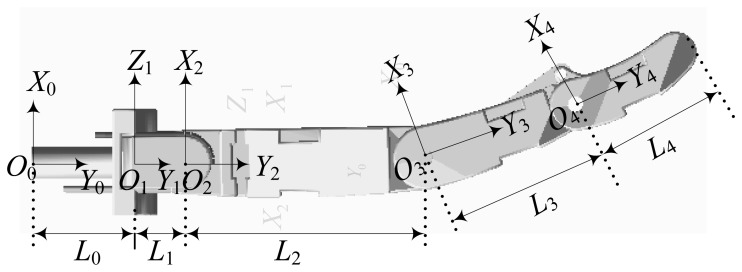
The coordinate systems buit in the finger model.

The Cartesian and joint coordinate systems are built, as show in Figure 7, where *O*_0_ is the Cartesian coordinate system; *O*_1_, *O*_2_, *O*_3_ and *O*_4_ are respectively the coordinate system of each joint.

The Denavit-Hartenberg(D-H) parameters of the robot finger are listed in Table 1.

where *η_i_*_−1_ represents the rotation angle round *Y_i_*_−1_ axis; *L_i_*_−1_ is defined as the distance from *Z_i_*_−1_ to *Z_i_*; *d_i_* is defined as the distance from *Y_i_*_-1_ to *Y_i_*.

According to Figure 7 and Table 1, the kinematics equation of the three-DOF finger is expressed as:

(12) $$A=A_{1}A_{2}A_{3}A_{4}A_{5}$$

where:

$$\begin{array}{cc} A_{1}=\left[\begin{array}{cccc} 1 & 0 & 0 & 0\\ 0 & \cos\theta_{1} & -\sin\theta_{1} & L_{0}\\ 0 & \sin\theta_{1} & \cos\theta_{1} & 0\\ 0 & 0 & 0 & 1 \end{array}\right] & A_{2}=\left[\begin{array}{cccc} \cos\theta_{2} & -\sin\theta_{2} & 0 & 0\\ \sin\theta_{2} & \cos\theta_{2} & 0 & L_{1}\\ 0 & 0 & 1 & 0\\ 0 & 0 & 0 & 1 \end{array}\right]\\ A_{3}=\left[\begin{array}{cccc} \cos\theta_{3} & -\sin\theta_{3} & 0 & 0\\ \sin\theta_{3} & \cos\theta_{3} & 0 & L_{2}\\ 0 & 0 & 1 & 0\\ 0 & 0 & 0 & 1 \end{array}\right] & A_{4}=\left[\begin{array}{cccc} \cos\theta_{4} & -\sin\theta_{4} & 0 & 0\\ \sin\theta_{4} & \cos\theta_{4} & 0 & L_{3}\\ 0 & 0 & 1 & 0\\ 0 & 0 & 0 & 1 \end{array}\right]\\ A_{5}=\left[\begin{array}{cccc} 1 & 0 & 0 & 0\\ 0 & 1 & 0 & L_{4}\\ 0 & 0 & 1 & 0\\ 0 & 0 & 0 & 1 \end{array}\right] & A=\left[\begin{array}{cccc} n_{x} & o_{x} & a_{x} & x\\ n_{y} & o_{y} & a_{y} & y\\ n_{z} & o_{z} & a_{z} & z\\ 0 & 0 & 0 & 1 \end{array}\right] \end{array}$$

where *x*, *y* and *z* are the coordinate values of the fingertip in Cartesian coordinate system respectively.

The following equations can be obtained from Equation (12):

(13) $$-y\sin\theta_{1}+z\cos\theta_{1}+L_{0}\sin\theta_{1}=0$$

(14) $${w_{1}}^{2}+{\left(w_{2}-L_{2}\right)}^{2}-2L_{3}(-w_{1}\sin\theta_{3}+(w_{2}-L_{2})\cos\theta_{3})+{L_{3}}^{2}={L_{4}}^{2}$$

(15) $${\left(q_{2}-L_{1}\right)}^{2}+{q_{1}}^{2}-2((q_{2}-L_{1})\cos\theta_{2}-q_{1}\sin\theta_{2})L_{2}+{L_{2}}^{2}={L_{4}}^{2}+{L_{3}}^{2}+2L_{4}L_{3}\cos\theta_{4}$$

(16) $$m_{1}\cos\theta_{4}+m_{2}\sin\theta_{4}-L_{3}\sin\theta_{4}=0$$

where:

$$\begin{array}{c} q_{1}=x,q_{2}=y\cos\theta_{1}+z\sin\theta_{1}-L_{1}\cos\theta_{1}\\ w_{1}=q_{1}\cos\theta_{2}+q_{2}\sin\theta_{2}-L_{1}\sin\theta_{2}\\ w_{2}=-q_{1}\sin\theta_{2}+q_{2}\cos\theta_{2}-L_{1}\cos\theta_{2}\\ m_{1}=w_{1}\cos\theta_{3}+w_{2}\sin\theta_{3}-L_{2}\sin\theta_{3}\\ m_{2}=-w_{1}\sin\theta_{3}+w_{2}\cos\theta_{3}-L_{2}\cos\theta_{3} \end{array}$$

**Figure 8.**
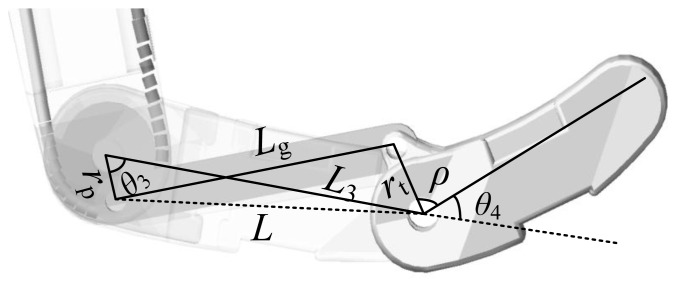
The coupled structure of the three-DOF finger.

In addition, there is relationship between *θ*_3_ and *θ*_4_ due to the coupled structure, as shown in Figure 8.

The relational expressions between *θ*_3_ and *θ*_4_ are given by:

(17) $$\begin{array}{c} L=\sqrt{{r_{\text{p}}}^{2}+{L_{3}}^{2}-2r_{\text{p}}L_{3}\cos\theta_{3}}\\ \alpha =ar\cos(\frac{{r_{t}}^{2}+L^{2}-{L_{g}}^{2}}{2r_{t}L})\\ \beta =\alpha -ar\cos(\frac{L^{2}+{L_{3}}^{2}-{r_{p}}^{2}}{2LL_{3}})\\ \theta_{4}=\pi -\rho -\beta \end{array}$$

where *ρ*, *r*_p_, *L*_g_ and *r*_t_ are constant, *ρ* = 1.52 rad, *r*_p_ = 4 mm, *L*_g_ = 27 mm, *r*_t_ = 6.2 mm.

It is easy to get the solution of *θ*_1_ from Equation (13). However, Equations (14–17) are nonlinear and the computation cost is high, which is a limitation of inverse kinematics [16]. Therefore it is not easy to get the solutions of *θ*_2_, *θ*_3_ and *θ*_4_. Since the control system is built in MATLAB, the fsovle function in MATLAB can be used to solve the nonlinear equations. The fsovle function solves the equations by the iterative method and hence the initial values of the iteration have a great influence on the simulation speed. Since the angular displacements are continuous, the current solutions can be used as the initial values of the next iteration.

### 3.4. The Position Controller

In order to control the position of the robot finger accurately, it is necessary to get the relationship between the joint angular displacements and the tendon displacements. Due to the particular design and neglecting the tendon elasticity, the relationship can be expressed as [9,10]:

(18) $$\Delta l=R^{\text{T}}\Delta \theta$$

where ***R*** is defined in Equation (3); Δ***l*** and Δ***θ*** are defined in Section 3.1.

## 4. Simulation and Analysis

### 4.1. Simulation Setups

**Figure 9.**
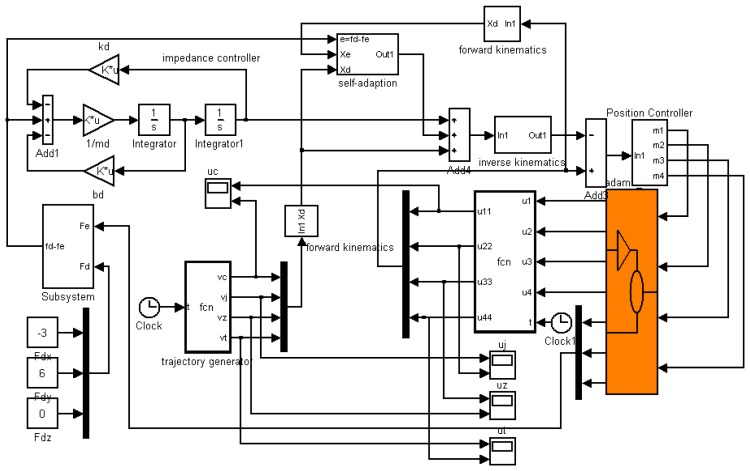
The Control system built in MATLAB.

The control system is built in MATLAB using the control module, as shown in Figure 9. The sample time of the simulation is 0.001 s. The simulation is mainly to validate the performance of the adaptive impedance control in uncertain environment and the reasonableness of the robot finger model. The impedance parameters and the adaptive parameters are given by:

$$\begin{array}{l} \begin{array}{lll} M_{\text{d}}=\left[\begin{array}{ccc} 200 & 0 & 0\\ 0 & 200 & 0\\ 0 & 0 & 200 \end{array}\right]\text{kg}, & K_{\text{d}}=\left[\begin{array}{ccc} 100 & 0 & 0\\ 0 & 100 & 0\\ 0 & 0 & 100 \end{array}\right]\text{N}/\text{m}, & B_{\text{d}}=\left[\begin{array}{ccc} 3000 & 0 & 0\\ 0 & 3000 & 0\\ 0 & 0 & 3000 \end{array}\right]N/(m/\text{s}) \end{array}\\ \sigma_{2}=0.001,\sigma_{1}=\sigma_{3}=0.09,\alpha_{1}=\beta_{1}=\lambda_{1}=0.1,\omega_{p}=0.27,\omega_{v}=0.25. \end{array}$$

### 4.2. The Position Control

**Figure 10.**
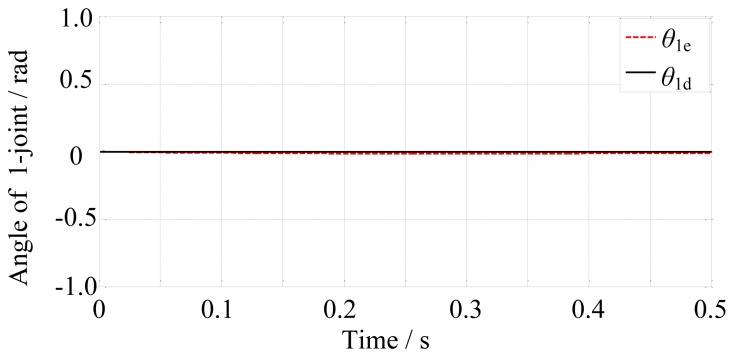
The desired and actual angular displacements of the 1-joint.

**Figure 11.**
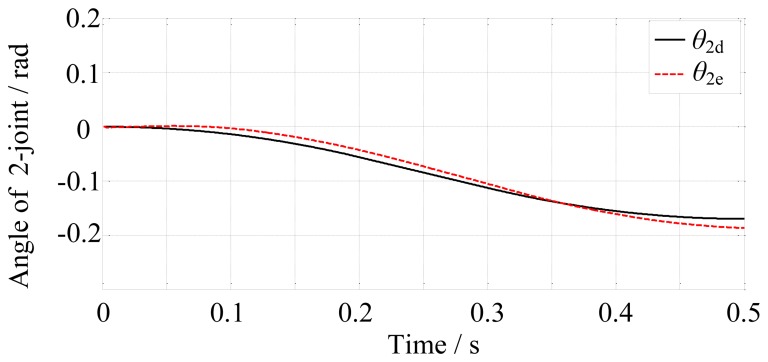
The desired and actual angular displacements of the 2-joint.

**Figure 12.**
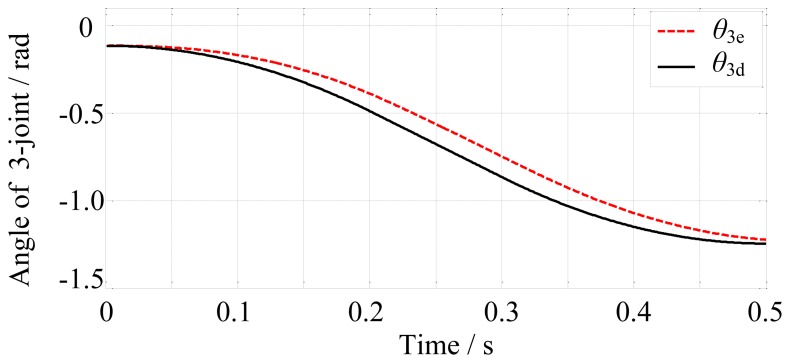
The desired and actual angular displacements of the 3-joint.

In the free space, the finger motion is controlled by the position controller. The simulation time is set to 0.5 s. The final-desired angular displacements of the joints are [0, −0.17, −1.25]^T^ rad. Then the position controller controls the finger motion according to the desired trajectories. The results are shown in Figures 10, 11 and 12.

From Figures 10, 12 and 12, we may conclude that the actual angular displacements lag behind the desired one at the beginning. The reason is that the finger is driven by tendons which have elasticity and it takes some time for the tendons to go from slack to tight in the simulation. So, on the whole, the results are satisfactory.

### 4.3. Uncertainties in Environment Stiffness

**Figure 13.**
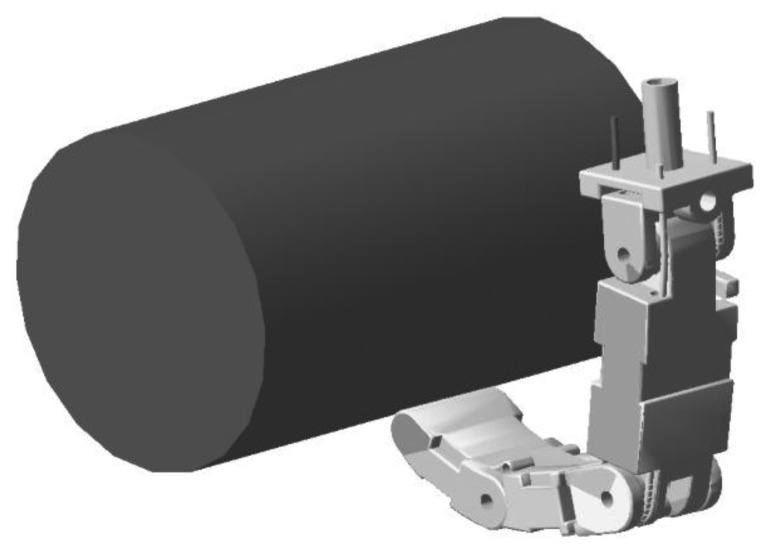
The finger makes contact with the environment in ADAMS.

**Figure 14.**
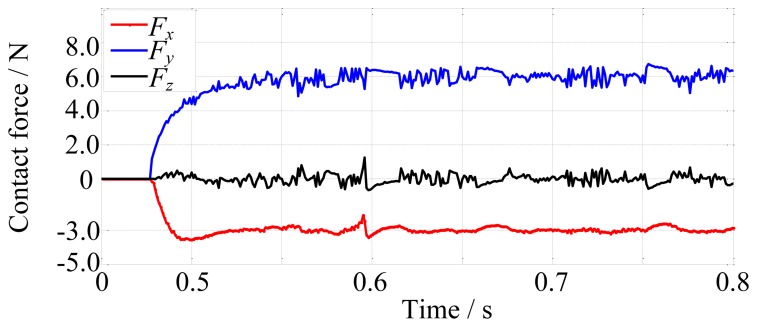
The force tracking results when environment stiffness is 10,000 N/m.

Firstly, the robot finger is required to track on the environment which is a cylinder and has the stiffness of 10,000 N/m. The desired contact force is [−3, 6, 0]^T^ N. The fingertip makes contact with the environment at about 0.48 s, as shown in Figure 13. The force tracking results are shown in Figure 14.

**Figure 15.**
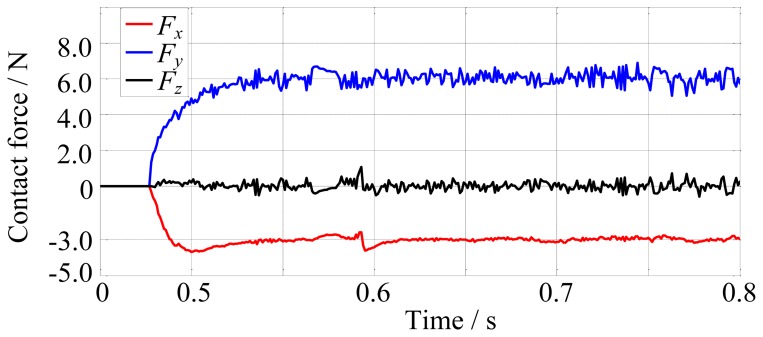
The force tracking results when environment stiffness is 80,000 N/m.

In the next simulation, the environment stiffness is modified as 80,000 N/m and other conditions remain unchanged. After rerunning, the force tracking results are shown in Figure 15.

Figures 14 and 15 show that the average error of the force tracking is both less than 0.5 N in different stiffness although the actual force is oscillating due to the tendons, so on the whole, the force tracking results are satisfying when the environment stiffness is different, that is to say, without knowing the environment stiffness information, the force control algorithm performs well.

### 4.4. Uncertainties in Environment Position

**Figure 16.**
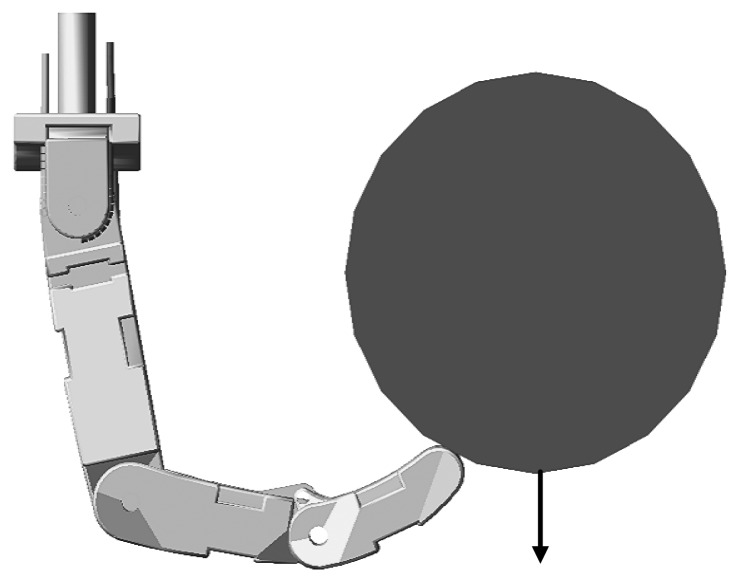
The direction of the row is the direction of the change.

**Figure 17.**
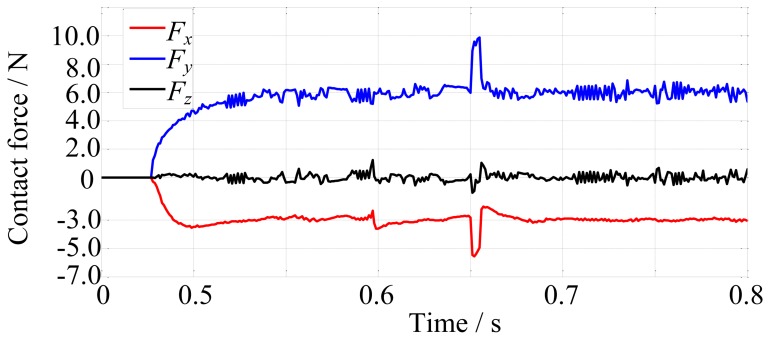
The force tracking results when environment position changed at 0.65 s.

In order to validate the robustness without knowing the environment position, let the environment position suddenly change at 0.65 s in the simulation. The change takes 0.005 s and the direction is shown in Figure 16. The displacement of the environment is 0.5 mm. The environment stiffness is 10,000 N/m and other conditions remain unchanged. The force tracking results are shown in Figure 17.

Comparing the force plot of Figure 17 with that of Figure 14 shows that the contact force changes at 0.65 s in Figure 17 due to the sudden change of environment position and the force overshoots are about 70% on *x*-direction and 65% on *y*-direction and the settling time is about 0.02 s. These demonstrate that without knowing the environment position information, the force control algorithm is robust.

**Figure 18.**
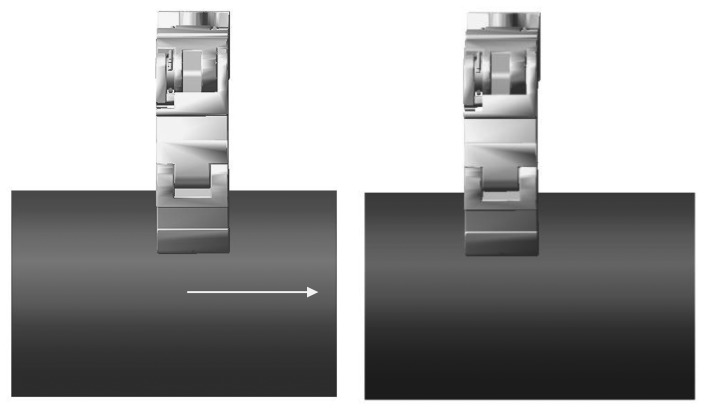
The finger makes contact with moving environment.

**Figure 19.**
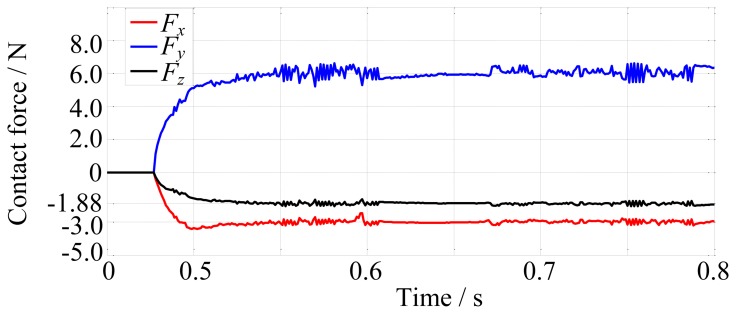
The force tracking results while the environment is moving.

Another simulation is carried out for the moving environment, as shown in Figure 18. The velocity of the environment movement is 0.025 m/s. There is dynamic friction between fingertip and environment due to the relative sliding. Hence, the desired force is set to [−3, 6, −1.88]^T^ N, where the force on *z*-direction is decided by friction coefficient. And the environment stiffness is also 10,000 N/m. The force tracking results are shown in Figure 19. Figure 19 shows that the environment movement has little impact on the force tracking results. This simulation demonstrates that the algorithm can perform diverse tasks.

## 5. Conclusions

In the joint simulation with ADAMS and MATLAB, the results of the position tracking and force tracking are satisfactory on the whole. In particular, the control system can keep the contact force near to expected value when the position or stiffness of the environment is uncertain, so the proposed adaptive impedance control algorithm is robust and the control system is suitable for a tendon-driven hand and the dexterous hand model built in ADAMS is reasonable. However, the tendon friction and joint friction are not considered in the robot hand model built in ADAMS. The friction can affect the control performance in practice, so the following research should build the friction model for the tendons and joints.

## Figures and Tables

**Table 1.** The D-H parameters of the robot finger.

***i***	***η****_i_***_−1_/(°)**	***L****_i_***_−1_/mm**	***d****_i_***/mm**	***θ****_i_***/rad**
1	90.0	21	0	*θ*_1_
2	−90.0	9	0	*θ*_2_
3	0	45	0	*θ*_3_
4	0	30	0	*θ*_4_
